# Analysis of different domains of physical activity with health-related quality of life in adults: 2-year cohort

**DOI:** 10.1186/s12955-022-01981-3

**Published:** 2022-04-29

**Authors:** Catarina Covolo Scarabottolo, William Rodrigues Tebar, Luis Alberto Gobbo, David Ohara, Aline Duarte Ferreira, Daniel da Silva Canhin, Diego Giulliano Destro Christofaro

**Affiliations:** 1 School of Technology and Sciences, Graduate Program in Movement Sciences, Physical Education Department, São Paulo State University (Unesp), Rua Roberto Simonsen, 305, Presidente Prudente, São Paulo CEP: 19060-900 Brazil; 2 Center of Clinical and Epidemiological Research, University Hospital, University of Sao Paulo, São Paulo, Brazil; 3 Department of Health Sciences, Santa Cruz State University, Ilhéus, Brazil; 4 Physiotherapy Department, University of Western São Paulo (UNOESTE), Presidente Prudente, Brazil; 5 School of Technology and Sciences, Graduate Program in Physiotherapy, Physiotherapy Department, São Paulo State University (Unesp), Presidente Prudente, Brazil

**Keywords:** Quality of life, Sedentary lifestyle, Physical activity, Mental health, Adults

## Abstract

**Background:**

It is estimated that, worldwide, 9% of deaths occur as a result of insufficient physical activity (PA) practice. Practicing PA can prevent and/or reduce the deleterious effects of different types of diseases and can improve general health aspects related to health-related quality of life (HRQoL).

**Purpose:**

To analyze the relationship of different PA domains with different HRQoL domains over a two-year period.

**Methods:**

This is an observational study with a two-year longitudinal design. The sample, composed of adults, was selected from a randomization of the streets of the different regions (north, south, east, west and center) covering individuals from all areas of the city. To assess the practice of PA, the Baecke questionnaire was used. The instrument Medical Outcomes Study SF-36-Item Short Form Health Survey was used to assess the HRQoL domains. Linear regression models were used to analyzed the association of different PA domains with changes in HRQoL. Multivariate statistical models were adjusted for gender, age, socioeconomic status, marital status, the respective PA score at baseline, smoking, body mass index, and morbidity.

**Results:**

331 adults were evaluated. There was a decrease in the practice of PA in the occupational domain and an increase in the leisure/locomotion domain. Regarding HRQoL, there was an increase in the scores of body pain and mental health, and a decrease in scores of general health, vitality, social aspects and emotional aspects. The PA practice in the occupational domain was inversely related to functional capacity (β = − 7.2 [CI 95% − 13.0; − 1.4]). The practice of PA through sports in leisure time was positively associated with vitality (β = 5.5 [CI 95% 0.2; 10.7]) and mental health (β = 15.2 [CI 95% 6.8; 23.7]). PA practice during leisure and locomotion was inversely associated with functional capacity (β = − 5.68 [CI 95% − 10.7; − 0.6]) and positively associated with vitality β = 4.8 [CI 95% 0.8; 8.7]) and mental health (β = 8.4 [CI 95% 2.0; 14.9]). The total PA practice was inversely associated with functional capacity (β = − 3.8 [CI 95% − 6.5; − 1.2]) and positively associated with pain in the body (β = 4.9 [CI 95% 0.3; 9.4]), vitality (β = 2.9 [CI 95% 0.7; 5.0]) and mental health (β = 5.7 [CI 95% 2.2; 9.2]).

**Conclusions:**

It is suggested that practicing PA continuously within a period of two years can positively affect some physical aspects and some mental aspects related to HRQoL, but not all of them. A strategy for public policy actions is to explore these variables by domain and thus detect the real needs and improvements that can be made for the population.

## Background

It is estimated that 9% of deaths in the world happen as a result of insufficient physical activity (PA) practice [1]. The worldwide prevalence of adults who do not meet the recommendations of the World Health Organization [2] to practice between 150 and 300 min of moderate to vigorous PA intensity is 28% [3, 4]. The highest prevalence of insufficient PA practice is found in Latin American and Caribbean countries, and, among them, Brazil has the highest prevalence of insufficient PA practice (47%) [5].

The practice of PA can contribute to the reduction of mortality rates and can represent a protective factor for the development of chronic non-communicable diseases (NCDs) such as cardiovascular diseases, high blood pressure, obesity, type 2 diabetes and some types of cancer [1, 6, 7]. In addition to NCDs, PA practice can improve aspects related to health-related quality of life aggregating physical and mental dimensions [8], such as the sense of well-being and general health perception [9].

One of the ways to reach the PA practice recommendations is to understand about the different domains in which PA can be practiced: occupational PA that occurs within the work environment through locomotion or by carrying weights; PA as a sport during leisure time, such as walking, weight training or sports in general; and finally, PA during leisure time or as a form of commuting. Practicing PA in different domains can prevent and reduce the risk of mortality from NCDs as observed in some studies [10, 11].

In addition to the physiological benefits that can be promoted by the practice of PA in different contexts, it is also believed in the benefits related to mental health [12–14] and consequently to the quality of life [15]. The concept of health-related quality of life (HRQoL) is widely used by both health professionals and the scientific community, and like the practice of PA, HRQoL can be explored in different domains covering physical, mental health and social aspects [16, 17].

However, the relationship between PA practice in different domains and HRQoL also analyzed in different domains is not clear in the literature. In a systematic review of the association of PA with quality of life in adults [18] the authors reported that most of the studies included in the review were cross-sectional, with more than 90% of the studies carried out in high-income countries (having an bias information in the literature referring to low and middle countries like Brazil) and most studies analyzed PA as a whole, not showing the specifics of each domain. More recently, in a systematic review and meta-analysis on this topic, the authors reported that 76% of the articles included in the study had a cross-sectional design, and, among the 23 longitudinal studies included in the study, 22 analyzed the leisure domain in isolation and only one study analyzed the different domains of PA practice [14].

Thus, the aim of the present study was to analyze the relationship of different domains of PA practice with different domains of HRQoL in a population of adults over a two-year period. Investigating these variables through information and population data obtained during a two-year period in the segment, seeking tools for both maintenance and increment of PA practice and HRQoL seems to be of great relevance in the current context of society.

## Methods

This is an observational study with a two-year longitudinal design, involving a randomized sample of adults. The study included non-institutionalized adults over 18 years of age, of both genders, living in an urban area in the city of Presidente Prudente—SP. The municipality of Presidente Prudente currently has an estimated population of 207,610 inhabitants, with a total of 176,124 inhabitants over the age of 18 living in urban areas, according to the Brazilian Institute of Geography and Statistics—IBGE. For data collection, all streets in the city of Presidente Prudente—SP were surveyed and the streets were divided according to neighborhood, postal code and geographic location between the North, South, East, West and Center. The planning and schedule of data collection were carried out using randomized lists of public places. Street randomization occurred according to the demand of the individuals interviewed, so that as many streets were drawn as needed to obtain a minimum sample in each region. In each of the selected streets, all existing households were visited. After explaining the research, individuals who met the inclusion criteria and voluntarily agreed to participate in this study were include in the sample.

As inclusion criteria for the study, the following were considered: (i) being 18 years old or older on the date of the first evaluation; (ii) not be institutionalized; (iii) does not have physical limitations that make it impossible for the participant to get up (eg, wheelchair users, bedridden); (iv) reside in the city of Presidente Prudente—SP for at least 2 years; (v) sign the Informed Consent Form. As an exclusion criterion from the study, the following were considered: (i) not having answered all the questions in the questionnaire.

**Fig. 1:**
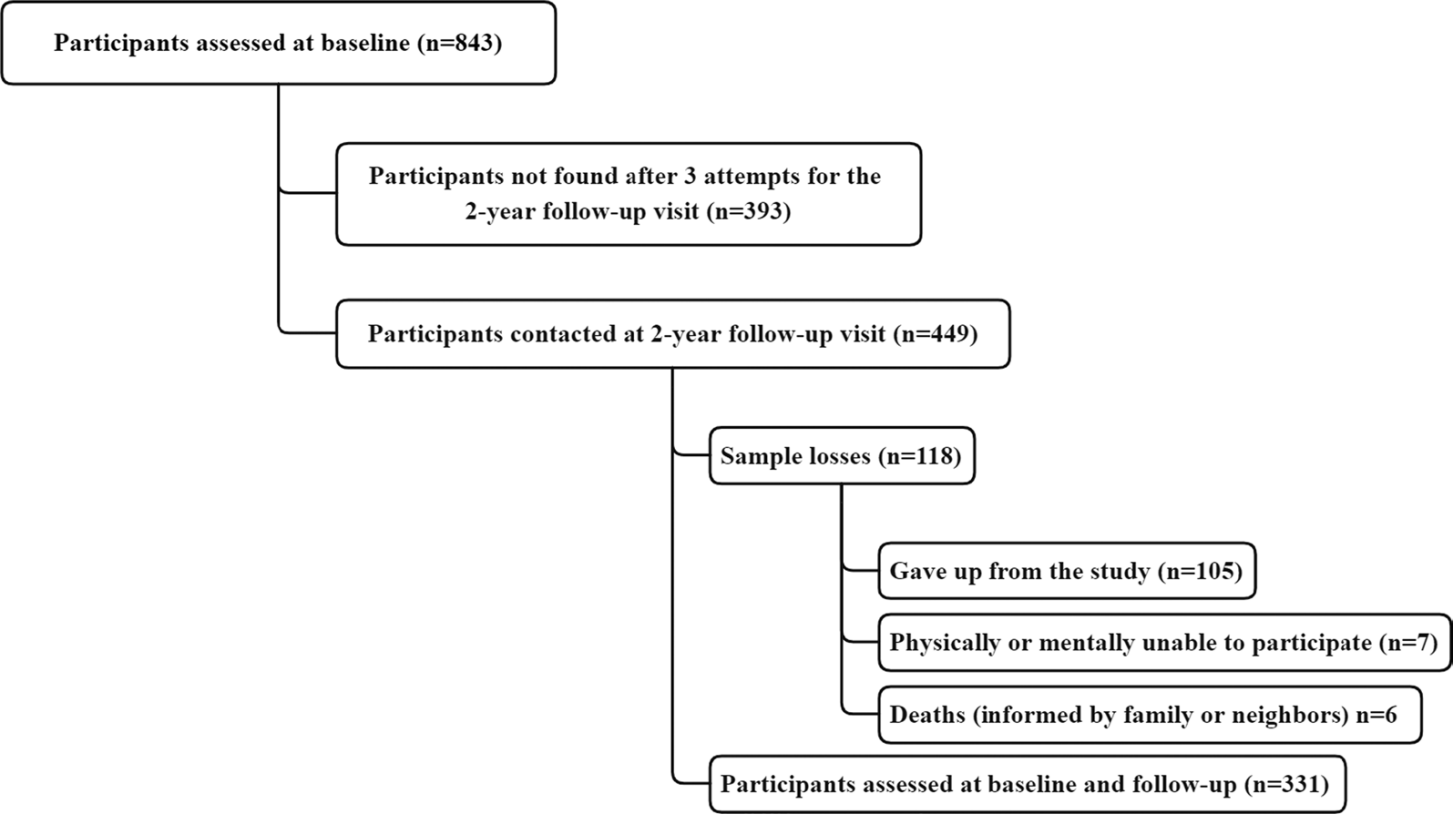
Flowchart of sampling process

The total period of data collection ranged from April 2016 to October 2019. Data were collected in face-to-face household interviews by previously trained evaluators and all assessments were carried out at the participant's home in a single day. The information collected was released on tablets through a digital interface developed in the Open Data Kit (ODK) application, which stores the data in a cloud and allows for export in a tab-delimited spreadsheet. Baseline data collection was carried out from March 2016 to August 2017, where 843 individuals were assessed according to a previous published sampling process [19]. Cohort data collection started 24 months after each participant's baseline collection, where the households of the evaluated participants were visited again and these, when found, were invited to perform the same study procedures again. Participants who were not found received two more attempts on separate days and were considered as not found (after three missed visits or moving to an unknown address). Participants who were contacted at follow-up visit, but did not participate in the follow-up assessment were classified as: (i) dropped out (participant refused to participate again in the research); (ii) unable (participant had a physical or psychological condition that prevented him/her from carrying out the research again); and (iii) death (report of a close person about his/her death—family members or neighbors). At the end of data collection, a total of 331 participants (observed sample power of 88.3% to predict changes in Health-related quality of life) were evaluated at both baseline and follow-up visits—Fig. 1.

This project was approved by the Research Ethics Committee of the Faculty of Science and Technology FCT/Unesp, Presidente Prudente Campus (CAAE protocol 45486415.4.0000.5402—06.19.2015).

### Physical activity (PA)

To assess the practice of PA, the questionnaire by Baecke et al. [20], previously validated for the Brazilian adult population [21], was used. This instrument assesses the usual practice of PA in the domains of occupation/work, leisure sports and free time/locomotion, through 16 questions on a Likert scale ranging from 1 to 5, generating a dimensionless score for each domain evaluated. Finally, the three scores are added to obtain the total PA score. Participants located in the 4th quartile of the total PA score were classified as “most active” and those located in the lower quartiles (Q1, Q2 and Q3) were classified as “less active”.

### Health related quality of life (HRQoL)

To assess HRQoL, the instrument Medical Outcomes Study SF-36-Item Short Form Health Survey (SF-36) was used. The SF-36 consists of 36 items covering eight domains about HRQOL: functional capacity, physical limitations, body pain, general health perception, vitality, social aspects, emotional limitations, mental health and the current perception of health in comparison a year ago. This instrument has a final score from 0 to 100, where 0 represents the worst score and 100 represents the best score in relation to HRQoL [17].

### Covariates

The variables of age, gender, socioeconomic status, marital status, smoking, morbidity, and body mass index were considered as covariates in the present study. To assess the socioeconomic status of the participants, the Brazil Economic Classification Criteria [22] was used. This instrument considers the level of education and the number of certain rooms and consumer products in the individual's household, classifying the sample into economic classes from the highest to the lowest: A, B1, B2, C1, C2, D-E. Socioeconomic status was categorized into high (A), medium (B1, B2, C1), and low (C2, D-E), according to the criteria established in the instrument. The marital status was self-reported by the participant among the possible answers: (i) single; (ii) married; (iii) separate/divorced; (iv) widowed; and (v) other (not specified). The smoking habit was assessed by the single question: “Do you currently smoke?”, responses were “Yes” or “No”. Participants who reported to have medical diagnosis of hypertension, diabetes, or dyslipidemia were classified as having morbidity. Body mass index was assessed though objective measurements of body weight and height, with participants barefoot and wearing light clothes, being classified as normal weight, overweight, and obesity, according to cutoff points proposed by World Health Organization [23].

### Statistical analysis

For the sample characterization, categorical variables were presented in absolute numbers and frequency and continuous variables were presented in mean, standard deviation, median, interquartile range, and minimum and maximum values for both at baseline and follow-up, with comparisons analyzed by Wilcoxon paired test. Data normality for linear regression analysis was supported by values of asymmetry and kurtosis between + 1 and − 1 of included variables, which were considered as acceptable to prove univariate normality, mainly among samples higher than n = 300, as the case of present study [24]. Linear regression models were used to analyzed the association of different PA domains with changes in HRQoL (delta values). The first multivariate statistical model was adjusted for gender, age, socioeconomic status, marital status, and the respective PA score at baseline (considering the analyzed domain). The second model was adjusted by variables included in Model 1 more smoking, body mass index, and morbidity. The statistical significance used was 5% and the confidence interval adopted was 95% in analysis performed by IBM® SPSS Statistical Package version 25.

## Results

The sample of the present study consisted of 331 adults with a mean age of 59.6 ± 17.3 years, being 68.3% of women. The participants were predominantly from medium socioeconomic status (74.6%), married (55.9%), non-smokers (85.5%), and with overweight/obesity (67.7%). More than half of sample had comorbidity (53.8%), being hypertension the most prevalent (46.8%). The descriptive characteristics of the sample is presented in Table 1.

**Table 1:** Descriptive characteristics of sample (n = 331)

Age (years), mean (SD)	59.6 (17.3)
Gender, n (%)	
Men	105 (31.7)
Women	226 (68.3)
Socioeconomic status, n (%)	
High	68 (20.5)
Medium	247 (74.6)
Low	16 (4.8)
Marital status, n (%)	
Single	58 (17.5)
Married	185 (55.9)
Separate/divorced	28 (8.5)
Widowed	51 (15.4)
Other	9 (2.7)
Body mass index	
Normal weight	107 (32.3)
Overweight	120 (36.3)
Obesity	104 (31.4)
Smoking, n (%)	
No	283 (85.5)
Yes	48 (14.5)
Morbidity, n (%)	178 (53.8)
Hypertension	155 (46.8)
Diabetes	57 (17.3)
Dyslipidemia	71 (21.4)

SD, Standard deviation

Table 2 shows the comparison between baseline vs. follow-up values of PA practice and HRQoL in different domains. It was observed a decrease in the practice of occupational PA and an increase in the practice of leisure/locomotion PA after a two-year follow-up. Considering the HRQoL, there was an increase in domains score of body pain and mental health, while there were a decrease in scores of general health, vitality, social aspects and emotional aspects HRQoL domains.

**Table 2:** Baseline vs. 2-year follow-up comparison of physical activity and health-related quality of life in adults (n = 331)

	Baseline			Follow-up			*p* value*
	Mean (SD)	Median (Q1–Q3)	Min./Max	Mean (SD)	Median (Q1–Q3)	Min./Max	
Physical activity, score							
Occupational PA	2.9 (0.7)	3.0 (2.5–3.5)	1.0–4.9	2.7 (0.5)	2.8 (2.4–3.0)	1.3–4.3	< 0.001
Leisure sports practice	1.9 (0.6)	1.8 (1.5–2.3)	1.0–4.3	2.0 (0.5)	2.0 (1.8–2.3)	1.0–4.0	0.068
Leisure and locomotion PA	2.0 (0.7)	2.0 (1.5–2.5)	1.0–4.8	2.2 (0.6)	2.3 (1.8–2.8)	1.0–4.0	< 0.001
Total PA	6.9 (1.4)	6.8 (5.9–7.6)	3.5–11.3	6.9 (1.2)	7.0 (6.1–7.6)	3.5–10.1	0.423
HRQoL, score							
Functional capacity	71.6 (27.7)	80.0 (55.0–95.0)	0.0–100.0	77.0 (12.0)	80.0 (72.0–80.0)	16.0–100.0	0.067
Physical limitation	72.6 (42.8)	100.0 (25.0–100.0)	0.0–100.0	77.0 (22.5)	85.0 (70.0–90.0)	0.0–100.0	0.259
Body pain	63.1 (26.8)	62.0 (51.0–84.0)	0.0–100.0	76.9 (41.1)	100.0 (75.0–100.0)	0.0–100.0	< 0.001
General health	68.3 (20.5)	72.0 (57.0–82.0)	0.0–100.0	59.8 (19.3)	62.0 (51.0–62.0)	0.0–100.0	< 0.001
Vitality	70.8 (19.9)	75.0 (60.0–85.0)	0.0–100.0	64.5 (16.6)	62.0 (57.0–75.0)	0.0–100.0	< 0.001
Social aspects	81.6 (23.7)	87.5 (75.0–100.0)	0.0–100.0	72.6 (14.3)	75.0 (65.0–80.0)	10.0–100.0	< 0.001
Emotional aspects	81.8 (37.5)	100.0 (100.0–100.0)	0.0–100.0	78.3 (17.1)	75.0 (75.0–100.0)	12.5–100.0	< 0.012
Mental health	72.6 (19.9)	76.0 (64.0–88.0)	8.0–100.0	88.7 (30.9)	100.0 (100.0–100.0)	0.0–100.0	< 0.001

PA, physical activity; SD, standard deviation; Q1–Q3, values from 1st and 3rd quartiles; Min./Max., minimum and maximum observed values

*Wilcoxon paired test

Table 3 presents the association of Occupational PA and leisure-time sports practice with domains of HRQoL. It is observed that after two years, occupational PA was inversely related to functional capacity, and for each increase in PA levels in this domain, there was a reduction of approximately 7 points in the functional capacity score. The continued practice of leisure sports PA was positively associated with greater vitality and mental health. For each increase in this type of PA, there were 15-point increments in the mental health score.

**Table 3:** Relationship of occupational physical activity and leisure-time sports practice with changes in different domains of health-related quality of life in adults (n = 331)

	Model 1		Model 2	
	β (95% CI)	*p* value	β (95% CI)	*p* value
**Occupational physical activity score**				
Δ Functional capacity	**−** **7.1 (−** **12.9; −** **1.3)**	**0.016**	**−** **7.2 (−** **13.0; −** **1.4)**	**0.015**
Δ Physical limitation	5.8 (− 4.2; 15.9)	0.253	5.9 (− 4.2; 16.0)	0.248
Δ Body pain	5.8 (− 4.2; 15.8)	0.255	5.6 (− 4.4; 15.6)	0.273
Δ General health	− 1.4 (− 7.0; 4.1)	0.618	− 1.5 (− 7.0; 4.0)	0.591
Δ Vitality	2.6 (− 2.1; 7.3)	0.281	2.6 (− 2.2; 7.3)	0.289
Δ Social aspects	− 1.5 (− 7.2; 4.2)	0.607	− 1.7 (− 7.4; 4.1)	0.570
Δ Emotional aspects	− 4.3 (− 13.2; 4.6)	0.342	− 4.3 (− 13.2; 4.7)	0.347
Δ Mental health	4.0 (− 3.7; 11.7)	0.305	4.0 (− 3.7; 11.7)	0.311
**Leisure-time sports practice score**				
Δ Functional capacity	− 5.9 (− 12.6; 0.9)	0.087	− 5.4 (− 12.1; 1.3)	0.116
Δ Physical limitation	7.5 (− 3.8; 18.8)	0.190	8.0 (− 3.4; 19.4)	0.168
Δ Body pain	8.6 (− 2.5; 19.7)	0.129	8.3 (− 2.9; 19.5)	0.148
Δ General health	− 0.1 (− 6.4; 6.1)	0.965	− 0.7 (− 6.9; 5.5)	0.820
Δ Vitality	**5.3 (0.1; 10.5)**	**0.045**	**5.5 (0.2; 10.7)**	**0.042**
Δ Social aspects	5.8 (− 0.5; 12.1)	0.071	6.2 (− 0.2; 12.5)	0.057
Δ Emotional aspects	− 2.9 (− 12.9; 7.0)	0.564	− 2.2 (− 12.3; 7.8)	0.660
Δ Mental health	**15.1 (6.7; 23.5)**	**< 0.001**	**15.2 (6.8; 23.7)**	**< 0.001**

Bold indicates statistical significant difference (*p* < 0.05)

Model 1: adjusted for gender, age, socioeconomic status, marital status, and respective physical activity score at baseline; Model 2: adjusted by variables of Model 1 more body mass index, smoking, and morbidity

The association of leisure-time/locomotion and total physical activity scores with domains of HRQoL is presented in Table 4. Leisure-time/locomotion PA was inversely associated with functional capacity and positively associated with vitality and mental health. Total PA practice was inversely related to functional capacity. Being more physically active over two years was positively associated with increments in the body pain domain score. Positive associations were also observed between total PA and the vitality and mental health domains.

**Table 4:** Relationship of leisure-time/locomotion and total physical activity with changes in different domains of health-related quality of life in adults (n = 331)

	Model 1		Model 2	
	β (95% CI)	*p* value	β (95% CI)	*p* value
**Leisure-time/locomotion physical activity score**				
Δ Functional capacity	**−** **6.0 (−** **11.1; −** **1.0)**	**0.018**	**−** **5.68 (−** **10.7; −** **0.6)**	**0.027**
Δ Physical limitation	5.3 (− 3.2; 13.7)	0.222	5.6 (− 2.9; 14.1)	0.195
Δ Body pain	7.8 (− 0.6; 16.2)	0.068	7.6 (− 0.9; 16.1)	0.079
Δ General health	**4.8 (0.2; 9.5)**	**0.042**	4.1 (− 0.6; 8.7)	0.084
Δ Vitality	**4.6 (0.7; 8.6)**	**0.021**	**4.8 (0.8; 8.7)**	**0.019**
Δ Social aspects	1.5 (− 3.3; 6.3)	0.542	1.7 (− 3.2; 6.5)	0.504
Δ Emotional aspects	− 2.3 (− 9.8; 5.3)	0.557	− 1.8 (− 9.4; 5.7)	0.634
Δ Mental health	**8.4 (2.0; 14.8)**	**0.010**	**8.4 (2.0; 14.9)**	**0.011**
**Total physical activity score**				
Δ Functional capacity	**−** **3.9 (−** **6.6; −** **1.3)**	**0.003**	**−** **3.8 (−** **6.5; −** **1.2)**	**0.005**
Δ Physical limitation	3.9 (− 0.6; 8.5)	0.091	4.1 (− 0.5; 8.7)	0.079
Δ Body pain	**5.0 (0.5; 9.6)**	**0.030**	**4.9 (0.3; 9.4)**	**0.037**
Δ General health	1.2 (− 1.3; 3.7)	0.361	0.8 (− 1.7; 3.3)	0.512
Δ Vitality	**2.8 (0.7; 4.9)**	**0.009**	**2.9 (0.7; 5.0)**	**0.009**
Δ Social aspects	1.2 (− 1.4; 3.8)	0.372	1.2 (− 1.4; 3.9)	0.352
Δ Emotional aspects	− 2.0 (− 6.0; 2.1)	0.343	− 1.7 (− 5.8; 2.3)	0.402
Δ Mental health	**5.7 (2.2; 9.1)**	**0.001**	**5.7 (2.2; 9.2)**	**0.001**

Bold indicates statistical significant difference (*p* < 0.05)

Model 1: adjusted for gender, age, socioeconomic status, marital status, and respective physical activity score at baseline; Model 2: adjusted by variables of Model 1 more body mass index, smoking, and morbidity

## Discussion

In the period of two years, a decrease in PA practice in the field of work/occupation was observed. On the other hand, there was an increase in the practice of PA in the leisure/movement domain. Regarding HRQoL, an increase in the scores of the domains of body pain and mental health was observed, showing an improvement in the perception of these domains by the participants. On the other hand, there was a decrease in HRQoL scores related to general health, vitality, social and emotional aspects.

The positive association between PA and physical health parameters have been previously observed in previous studies [25, 26]. On the other hand, some researchers have reported that PA in the occupational domain was inversely related to some domains of HRQoL such as body pain and mental health [27], but not with the domain of functional capacity. Thus, it is speculated that the practice of PA in the occupational domain is composed of repetitive activities and/or movements, often performed unilaterally and therefore may negatively impact the functional capacity of individuals. Until a few years ago, to our knowledge, there was little evidence in the literature investigating the relationship of PA in the occupational domain and HRQoL parameters [27], and few studies were longitudinal [28] and most studies have analyzed the relationship between leisure-time physical activity and HRQoL [29, 30]. Some authors suggest that, in certain occupations, walking in the work environment can be a strategy to reduce psychological distress [31] and consequently improve mental health. In this sense, the practice of occupational PA should not be completely disregarded and future studies should investigate different types of occupations, as well as potential mechanisms and moderators that can influence these variables.

Leisure-time sports practice was positively associated with the vitality and mental health domains of the HRQoL. Evidence about the benefits that the practice of PA in leisure has on physical and mental health is consistent in the scientific literature [31, 32]. Previous studies have reported positive associations between the vitality and mental health domains of HRQoL and PA in sports practices during leisure time, in line with our findings [25, 30]. Vitality encompasses an individual's perception of energy and/or fatigue, while the mental health domain encompasses perceptions of suffering and/or psychological well-being [17]. Thus, it is believed that practicing sports PA during leisure time can positively impact HRQoL, especially in the domains involving vitality and mental health. Furthermore, leisure physical activities are generally those in which practitioners choose for greater affinity and that can provide pleasure [14], which would generate a feeling of satisfaction and successively higher mental health scores.

Leisure and locomotion PA was inversely associated with functional capacity, as observed by Jurakic et al. [27]. Some possible reasons for this finding can be speculated. The inadequate infrastructure of streets and sidewalks can make it difficult and/or harm the individual's movement and, consequently, have a negative impact on functional capacity. In this sense, socioeconomic differences and inequalities, reflections of current society, can directly influence aspects related to quality of life during leisure time commuting [33].

On the other hand, the domain of PA in leisure and locomotion was positively associated with general health, vitality and mental health. Ribeiro et al. [34] corroborate our findings, in which active commuting was the domain that was most related to better aspects of HRQoL (among them: vitality, social aspects and mental health). Other studies have also reported positive associations between this PA domain and aspects related to HRQoL [15, 35], strengthening evidence that practicing PA during leisure and locomotion can benefit related health aspects both the physical part as the mental, considering that they are domains that encompass physical and mental concepts.

When PA was analyzed in general, an inverse association with functional capacity was observed. To the best of our knowledge, studies reporting similar results have not been observed before. However, previous studies have reported positive relationships with PA practice and functional capacity [25, 26]. One issue that could possibly contribute to discrepancies between studies and make comparisons difficult is the use of different methods employed, emphasizing that most studies are cross-sectional, preventing analysis of cause-and-effect relationships. In addition, both PA and HRQoL measures were assessed through self-report and, therefore, the possibility of misclassification cannot be ruled out.

Total PA, covering the three domains together, was positively associated with better HRQL scores, showing a better perception by the participants in the following HRQoL domains: body pain, vitality and mental health. Previous studies that analyzed PA as a whole reported similar results [36, 37]. In this sense, increasing the practice of PA in all domains, resulting in a higher total PA score, can play an important role in increasing both the scores of these three HRQoL domains and the others.

The present study has some limitations. The instrument used to assess the practice of PA, even though it is an instrument widely used by the scientific community and validated for the study population, does not provide information on the duration or intensity of the practice of PA. In addition, the lack of information about symptoms of anxiety and depression may compromise these findings. The results must be interpreted with caution, as the study was conducted in a city in Brazil, and cannot be extrapolated to other regions or countries, considering that both the practice of PA in each domain and the perception of HRQoL could be influenced by the context cultural aspects that each individual is inserted in and sociodemographic and climatic factors. The present study had a substantial sample loss, where the baseline participants who were not found for the second visit showed significant differences in the age (59.7 vs. 54.5, *p* = 0.001) and proportions of gender (*p* = 0.001) and marital status (*p* = 0.031), which could be influenced the representativeness of the sample and limited the results. On the other hand, as positive and advance points of the study, its longitudinal design stands out, preventing the effect of reverse causality. The randomly selected sample prevents homogeneities likely to occur in samples selected for convenience. Furthermore, analyzes adjusted for confounding factors that may influence the study variables represent an important point of the present study.

## Conclusions

It is concluded that the continuity of PA practice in each domain over a two-year period was associated in a distinct way with the HRQoL domains. The results of the present study suggest that practicing PA continuously within a period of two years can positively impact some physical aspects and some mental aspects related to HRQoL, but not all of them. Also, some PA practice domains can negatively impact some HRQoL domains. Thus, exploring PA by domains and not just in its entirety can represent an important strategy to increase PA practice, both for actions aimed at public policies, and to inform the population and consequently improve domains related to HRQoL, an important focus in current times.

## Data Availability

All data generated or analysed during this study are included in this.

## References

[1] Lee I-M, Shiroma EJ, Lobelo F, Puska P, Blair SN, Katzmarzyk PT (2012). Effect of physical inactivity on major non-communicable diseases worldwide: an analysis of burden of disease and life expectancy. Lancet (London, England).

[2] World Health Organization (2020). WHO guidelines on physical activity and sedentary behaviour: at a glance.

[3] Bull FC, Al-Ansari SS, Biddle S, Borodulin K, Buman MP, Cardon G (2020). World Health Organization 2020 guidelines on physical activity and sedentary behaviour. Br J Sports Med.

[4] DiPietro L, Al-Ansari SS, Biddle SJH, Borodulin K, Bull FC, Buman MP (2020). Advancing the global physical activity agenda: recommendations for future research by the 2020 WHO physical activity and sedentary behavior guidelines development group. Int J Behav Nutr Phys Act.

[5] Guthold R, Stevens GA, Riley LM, Bull FC (2018). Worldwide trends in insufficient physical activity from 2001 to 2016: a pooled analysis of 358 population-based surveys with 1·9 million participants. Lancet Glob Health.

[6] World Health Organization (2010). Global recommendations on physical activity for health.

[7] Mok A, Khaw K-T, Luben R, Wareham N, Brage S (2019). Physical activity trajectories and mortality: population based cohort study. BMJ.

[8] Marcos-Delgado A, Fernández-Villa T, Martínez-González MÁ, Salas-Salvadó J, Corella D, Castañer O (2020). The effect of physical activity and high body mass index on health-related quality of life in individuals with metabolic syndrome. Int J Environ Res Public Health.

[9] Scarabottolo CC, Cyrino ES, Nakamura PM, Tebar WR, Canhin DS, Gobbo LA (2019). Relationship of different domains of physical activity practice with health-related quality of life among community-dwelling older people: a cross-sectional study. BMJ Open.

[10] Autenrieth CS, Baumert J, Baumeister SE, Fischer B, Peters A, Döring A (2011). Association between domains of physical activity and all-cause, cardiovascular and cancer mortality. Eur J Epidemiol.

[11] Wanner M, Tarnutzer S, Martin BW, Braun J, Rohrmann S, Bopp M (2014). Impact of different domains of physical activity on cause-specific mortality: a longitudinal study. Prev Med (Baltim).

[12] Kelly P, Williamson C, Niven AG, Hunter R, Mutrie N, Richards J (2018). Walking on sunshine: scoping review of the evidence for walking and mental health. Br J Sports Med.

[13] Mc Dowell CP, Carlin A, Capranica L, Dillon C, Harrington JM, Lakerveld J (2020). Associations of self-reported physical activity and anxiety symptoms and status among 7,874 Irish adults across harmonised datasets: a DEDIPAC-study. BMC Public Health.

[14] White RL, Babic MJ, Parker PD, Lubans DR, Astell-Burt T, Lonsdale C (2017). Domain-specific physical activity and mental health: a meta-analysis. Am J Prev Med.

[15] Pucci G, Reis RS, Rech CR, Hallal PC (2012). Quality of life and physical activity among adults: population-based study in Brazilian adults. Qual Life Res.

[16] Hart PD, Kang M, Weatherby NL, Lee YS, Brinthaupt TM (2015). Systematic review of health-related quality of life assessments in physical activity research. World J Prev Med.

[17] Ware JEJ, Sherbourne CD (1992). The MOS 36-item short-form health survey (SF-36). I. Conceptual framework and item selection. Med Care.

[18] Pucci GCMF, Rech CR, Fermino RC, Reis RS (2012). Association between physical activity and quality of life in adults. Rev Saude Publica.

[19] Canhin DS, Tebar WR, Scarabottolo CC, Silva GCR, Pinto RZ, Gobbo LA (2021). Physical activity across life stages and sleep quality in adulthood—an epidemiological study. Sleep Med.

[20] Baecke JA, Burema J, Frijters JE (1982). A short questionnaire for the measurement of habitual physical activity in epidemiological studies. Am J Clin Nutr.

[21] Florindo AA, Latorre MDRDDO, Jaime PC, Tanaka T, Zerbini CADF (2004). Methodology to evaluation the habitual physical activity in men aged 50 years or more. Rev Saude Publica.

[22] Associação Brasileira de Empresas de Pesquisa - ABEP. Critério de Classificação Econômica Brasil [Internet]. ABEP - Critério Brasil. 2009. http://www.abep.org/criterio-brasil

[23] Physical status: the use and interpretation of anthropometry. Report of a WHO Expert Committee. Vol. 854, World Health Organization technical report series. Switzerland; 1995. 8594834

[24] Mishra P, Pandey CM, Singh U, Gupta A, Sahu C, Keshri A (2019). Descriptive statistics and normality tests for statistical data. Ann Card Anaesth.

[25] Balboa-Castillo T, León-Muñoz LM, Graciani A, Rodríguez-Artalejo F, Guallar-Castillón P (2011). Longitudinal association of physical activity and sedentary behavior during leisure time with health-related quality of life in community-dwelling older adults. Health Qual Life Outcomes.

[26] Bertheussen GF, Romundstad PR, Landmark T, Kaasa S, Dale O, Helbostad JL (2011). Associations between physical activity and physical and mental health—a HUNT 3 study. Med Sci Sports Exerc.

[27] Jurakic D, Pedisic Z, Greblo Z (2010). Physical activity in different domains and health-related quality of life: a population-based study. Qual Life Res.

[28] Bize R, Johnson JA, Plotnikoff RC (2007). Physical activity level and health-related quality of life in the general adult population: a systematic review. Prev Med (Baltim).

[29] Vuillemin A, Boini S, Bertrais S, Tessier S, Oppert J-M, Hercberg S (2005). Leisure time physical activity and health-related quality of life. Prev Med (Baltim).

[30] Wendel-Vos GCW, Schuit AJ, Tijhuis MAR, Kromhout D (2004). Leisure time physical activity and health-related quality of life: cross-sectional and longitudinal associations. Qual life Res an Int J Qual life Asp Treat care Rehabil.

[31] White RL, Bennie J, Abbott G, Teychenne M (2020). Work-related physical activity and psychological distress among women in different occupations: a cross-sectional study. BMC Public Health.

[32] Saint-Maurice PF, Coughlan D, Kelly SP, Keadle SK, Cook MB, Carlson SA (2019). Association of leisure-time physical activity across the adult life course with all-cause and cause-specific mortality. JAMA Netw Open.

[33] Beenackers MA, Kamphuis CBM, Giskes K, Brug J, Kunst AE, Burdorf A (2012). Socioeconomic inequalities in occupational, leisure-time, and transport related physical activity among European adults: a systematic review. Int J Behav Nutr Phys Act.

[34] Ribeiro FE, Tebar WR, Vanderlei LCM, Fregonesi CEPT, Caldeira DT, Tosello G (2021). Physical activity domains are differently related with quality of life in breast cancer survivors: a cross-sectional study. Menopause.

[35] Andersen LB, Schnohr P, Schroll M, Hein HO (2000). All-cause mortality associated with physical activity during leisure time, work, sports, and cycling to work. Arch Intern Med.

[36] Heesch KC, van Gellecum YR, Burton NW, van Uffelen JGZ, Brown WJ (2015). Physical activity, walking, and quality of life in women with depressive symptoms. Am J Prev Med.

[37] Koolhaas CM, Dhana K, van Rooij FJA, Schoufour JD, Hofman A, Franco OH (2018). Physical activity types and health-related quality of life among middle-aged and elderly adults: the Rotterdam study. J Nutr Health Aging.

